# Effect of norbixin-based poly(hydroxybutyrate) membranes on the tendon repair process after tenotomy in rats^1^

**DOI:** 10.1590/s0102-865020190110000001

**Published:** 2020-01-13

**Authors:** Lízia Daniela e Silva Nascimento, Renata Amadei Nicolau, Antônio Luiz Martins Maia, José Zilton Lima Verde Santos, Khetyma Moreira Fonseca, Danniel Cabral Leão Ferreira, Rayssilane Cardoso de Sousa, Vicente Galber Freitas Viana, Luiz Fernando Meneses Carvalho, José Figueredo-Silva

**Affiliations:** I Fellow PhD degree, Postgraduate Program in Biomedical Engineering, Universidade do Vale do Paraíba (UNIVAP), Sao Jose dos Campos-SP. Assistant Professor of Kinesiology, MSc, Health Sciences Center, Physical Therapy, Universidade Estadual do Piauí (UESPI), Teresina-PI, Brazil. Conception, design, intellectual and scientific content of the study; acquisition and interpretation of data; technical procedures; manuscript preparation; Universidade Estadual do Piauí, Health Sciences Center, Physical Therapy, Teresina, PI, Brazil; II PhD, Collaborator, Postgraduate Program in Biomedical Engineering of the Research and Development Institute, UNIVAP, Sao Jose dos Campos-SP, Brazil. Conception, design, intellectual and scientific content of the study; interpretation of data; critical revision; III Associate Professor of Physiology, Health Sciences Center, UESPI, Teresina-PI, Brazil. Technical procedures; IV MSc, Assistant Professor, Center of Health Sciences, Medicine and Physical Therapy, UESPI, Teresina-PI. Brazil. Histopathological examinations; V Fellow PhD degree, Postgraduate Program in Pharmacology, Universidade Federal do Ceará (UFC), Fortaleza-CE. MSc in Biotechnology, Northeast Biotechnology Network - RENORBIO, Teresina-PI, Brazil. Acquisition of data, statistical analysis; VI Fellow Master degree, Professional Master's Program in Human and Animal Biotechnology MPBiotec, UESPI, Teresina-PI, Brazil. Technical procedures; VII Fellow PhD degree, Postgraduate Program in Biotechnology, Universidade Federal do Piauí (UFPI), Teresina-PI, Brazil. Membrane production; VIII PhD, Associate Professor, Postgraduate Program in Material Engineering (PPGEM), Federal Institute of Education, Science and Technology of Piaui, IFPI, Teresina-PI, Brazil. Membrane production; IX PhD, Full Professor, Postgraduate Program in Material Engineering (PPGEM), Federal Institute of Education, Science and Technology of Piaui, IFPI, Teresina-PI, Brazil. Membrane production; X PhD, Associate Professor of Pathology, Health Sciences Center, UESPI, Teresina-PI, Brazil. Histopathological examinations, critical revision

**Keywords:** Achilles Tendon, Collagen, Rats

## Abstract

**Purpose:**

To determine the efficacy of norbixin-based poly(hydroxybutyrate) (PHB) membranes for Achilles tendon repair.

**Methods:**

Thirty rats were submitted to total tenotomy surgery of the right Achilles tendon and divided into two groups (control and membrane; n = 15 each), which were further subdivided into three subgroups (days 7, 14, and 21; n = 5 each). Samples were analyzed histologically.

**Results:**

Histological analysis showed a significant reduction in inflammatory infiltrates on days 7, 14 (p < 0.0001 for both), and 21 (p = 0.0004) in the membrane group compared to that in the control group. There was also a significant decrease in the number of fibroblasts in the control group on days 7, 14 (p < 0.0001), and 21 (p = 0.0032). Further, an increase in type I collagen deposition was observed in the membrane group compared to that in the control group on days 7 (p = 0.0133) and 14 (p = 0.0107).

**Conclusion:**

Treatment with norbixin-based PHB membranes reduces the inflammatory response, increases fibroblast proliferation, and improves collagen production in the tendon repair region, especially between days 7 and 14.

## Introduction

Rupture of the Achilles tendon causes loss of tissue continuity and is a serious injury that mainly occurs during sports^1^. This injury primarily occurs in males between 30 and 50 years of age and affects not only professional athletes but also recreational athletes^2^. Further, it is associated with a high incidence (mean of 18 of 100.000 people)^3^, can cause severe reductions in the function of the ankle, and is characterized by pain, decreased range of motion, and delayed return to sports and work activities^4^. The treatment options for this lesion are conservative or surgical, with the latter being more frequently chosen as it restores tension and has a lower recurrence rate of rupture, which typically occurs at the same site as the first lesion^5^.

Achilles tendon rupture can be attributed to the combination of tendon degradation and failures in the inhibitory mechanism of the musculotendinous unit. The typical degenerative histological features of tendon rupture include changes in fiber structure, fiber arrangement, vascularization, cell morphology and cell proliferation. These alterations are more pronounced with acute ruptures than with symptomatic tendinopathies. Although augmentation or reconstructive procedures are used for tendon repair after injuries due to the extensive debridement required, acute Achilles tendon ruptures can also be candidates for surgery due to underlying tissue injury, lesion extension, and the activity of the affected population^6^.

The tendon tissue is a dense connective tissue and its function is to transmit the force of a muscle to a bone. The cells that compose the tendon tissue are tenoblasts (fibroblasts) combined with endothelial cells and some chondrocytes located in compression areas. The fibroblasts have different morphologies, lengths, and widths and decrease in number with aging, giving rise to tenocytes with greater diameters. These fibroblasts synthesize extracellular matrix (ECM) molecules such as collagen, which is the main structural protein of this matrix, in addition to proteoglycans and other proteins and is thus one of the most important cell types involved in the tendon repair process^7^ ^,^ ^8^.

For acute lesions, tendon tissue repair occurs in three phases, which involve distinct cellular and molecular cascades, including inflammation, proliferation, and remodeling. The inflammatory phase starts with the accumulation and coagulation of blood at the site, which causes the release of proinflammatory cytokines and growth factors by platelets to attract neutrophils, monocytes, and macrophages. Locally-secreted angiogenic factors trigger the formation of a vascular network to nourish the newly formed fibrous tissue. In the proliferation phase, fibroblasts are recruited and abundantly synthesize ECM components including randomly-arranged proteoglycans and type III collagen. In addition to fibroblasts, the number of endothelial cells and keratinocytes also increases. The remodeling phase is characterized by decreased cellularity, reduced matrix synthesis, decreased type III collagen production, and increased type I collagen generation. Fibers of this type of collagen start to organize along the axis of the tendon. Tendon cell proliferation and the synthesis of a new matrix are essential for the recovery of the tendon and the linear organization of collagen fibers, and tendon cells are restored during the remodeling stage. Type I collagen provides mechanical resistance for the tendon tissue and type III collagen plays an important role in the healing process^8^ ^,^ ^9^.

New biomaterials such as biological membranes have been developed to promote tissue repair. Accordingly, several scientific studies have been carried out to analyze their interactions with biological tissues in animals and the results were promising^10^ ^,^ ^11^ This has led to great progress in the total or partial repair of organs or tissues, such as in the case of norbixin-based poly(hydroxybutyrate) (PHB) membranes^12^.

PHB is a natural polymer that is generated from the activities of microorganisms such as fungi, algae and, bacteria from renewable carbon sources such as sugar cane. Moreover, it is a biodegradable thermoplastic. Since it is biocompatible, it is easily absorbed by the human body and can thus be used to produce biological membranes to guide and stimulate repair and regeneration^12^ ^,^ ^13^.

Combined with PHB for the production of membranes, natural products such as norbixin can provide important properties in the context of tissue healing. Norbixin is a water-soluble carotenoid obtained from the surface of annatto seeds of the plant species *Bixa orellana* L. This tropical plant is native to the forests of Central and South America^14^ ^,^ ^15^. The compounds extracted from *Bixa orellana* L. are widely used in many pharmaceutical, food, and cosmetic industries for their antibacterial, antifungal, and anti-inflammatory activities. Further, due to its antioxidant, antimicrobial, and antitumor properties, norbixin has great potential for use in biodegradable and biocompatible polymer membranes such as PHB^12^ ^,^ ^16^ ^,^ ^17^. Thus, considering the gap in the literature on the interaction of this biomaterial with biological tissues, the objective of this study was to evaluate the effect of norbixin-based PHB membranes on the Achilles tendon repair process after tenotomy in terms of the progression of the inflammatory process and the induction of type I and III collagen production.

## Methods

The protocol of this study was submitted for analysis and approved by the Ethics Committee for Animal Experimentation, Universidade Estadual do Piauí (UESPI), under the number 14776/16.

Experiments used 30 clinically-healthy male Wistar rats (*Rattus norvegicus*), of approximately 10 weeks of age and weighing between 250 and 300 g, obtained from the Animal House of the Center for Research in Biotechnology and Biodiversity, UESPI (NPBIO). All rats were maintained in polypropylene cages with feed and water *ad libitum* under controlled conditions of temperature (19ºC) and light (12–12-h light–dark cycles). The animals were divided into the control group (C) and the PHB membrane group (M), which were further subdivided into subgroups (C1, M1, C2, M2, C3, M3), each containing five animals, based on tissue collection at three different times, specifically 7, 14, and 21 days.

### Membrane preparation

The norbixin-based PHB membrane was previously prepared in the Master of Materials Engineering course at the Instituto Federal do Piauí and is under patent analysis with process number BR 10 2017 028640 1.

### Surgical procedure

All animals received pre-treatment with atropine (pre-analgesic) at a dose of 0.04 mL per 100 g of body weight, followed by a 15-min rest before the anesthetic procedure. The anesthetic drug was administered intramuscularly using 10% ketamine at a dose of 0.1 mL per 100 g of body weight and 2% xylazine at a dose of 0.1 mL per 100 g using a 1-mL insulin syringe for each animal. Next, the skin was trichotomized and cleaned with 2% iodinated alcohol in the Achilles tendon region of the animal's right paw.

The surgical procedure started with a longitudinal skin incision in the posterior region of the rat's right hind paw. The tendon was exposed and sectioned transversally in the middle region with a scalpel between the tendon insertion and the myotendinous junction in all animals, according to the protocol performed by Casalechi *et al.* ^18^. For all animals in the membrane group (M1, M2, and M3; n = 15), a membrane fragment of approximately 6 ′ 3 mm in length and width, respectively, was glued with methacrylate at two sites joining the two portions of the tendon. This included a site on the upper portion and another on the lower portion of the sectioned tendon, and both sites were located at the longitudinal and deep face of the Achilles tendon. The animals in the control groups (C1, C2, and C3; n = 15) did not receive the membrane or any material to fix the tendon stumps and their paws were not immobilized, as performed by Casalechi *et al.* ^18^, Moura Júnior *et al.* ^19^, and Parafioriti *et al.* ^20^. Subsequently, tenotomy was performed and the skin was closed with a 4-0 silk monofilament suture. After the surgical procedure, group C (C1, C2, and C3) and group M (M1, M2, and M3) rats were maintained in cages in groups of five animals. The animals in each cage received two drops (50 mg/kg) of dipyrone by gavage every 12 h for analgesia during treatment, according to the protocol adopted by Alves *et al.* ^21^.

### Euthanasia

The animals were euthanized on the 7th (subgroups C1 and M1), 14th (subgroups C2 and M2), and 21st (subgroups C3 and M3) days after the surgical procedure through the administration of an overdose of anesthetic, according to the Brazilian College of Animal Experimentation (COBEA, 1991). Next, the injured tendons were removed by dissecting the region from the Achilles insertion to the myotendinous junction, fixed in 10% formalin solution, and subjected to histological processing.

### Histological analysis

After fixation for 24 h, the samples were dehydrated in a series of alcohol solutions of increasing concentrations (70%, 80%, and 90%) and then diaphanized with xylol and later embedded in paraffin. For each block, four longitudinal semi-serial histological sections with a thickness of 5 μm were obtained and stained with hematoxylin & eosin (HE, n = 2) and Picrosirius Red (n = 2). The slides were analyzed using a trinocular microscope (Olympus^®^ CX31, Japan) with a 10× objective lens coupled with a digital camera (Bell & Howell, EU 16.0 Plus, U.S.A.) at 16× magnification. A Zeiss microscope with a 20× objective lens was used for the quantification of inflammatory cells and fibroblasts. The microphotographs were taken with a coupled Zeiss camera at 0.5× magnification and 5 mb resolution. The slides stained with Picrosirius Red were examined and photographed with a Leica^®^ DM 2000 polarized light microscope coupled with a DFC camera using a 10× objective lens.

The Image-J^®^ (Version 1.32 for Windows) computer image analysis program (from the National Institute of Health, NIH, Bethesda, USA) was used to quantify inflammatory cells and fibroblasts. Five fields per slide were evaluated at 200× magnification with the manual cell nucleus counting tool. The mean score obtained in each field was used to determine the unique score for each sample of each experimental group.

For the slides stained with Picrosirius Red, the amounts of type I and type III collagen were analyzed. The Image J Pro-plus program was used for quantification. After calibration, the two types of collagen were identified by their respective stains and the program was used to determine the percentage of type I and type III collagen in relation to the total area of the image.

### Statistical analysis

The data were evaluated based on the coefficient of variation and sample distribution to determine the statistical test using GraphPad Prism software, version 5.0 (GraphPad, California, USA), applying the Kolmogorov-Smirnov test. As the data were non-parametric, the Mann-Whitney test (intergroup analysis) and the Kruskal-Wallis test with Dunn's post-test (intragroup analysis) were used, considering a confidence interval of 95% and a significance level of 5% (p < 0.05).

## Results

### Histopathological analysis

#### Seven-day groups

**Figure 1:**
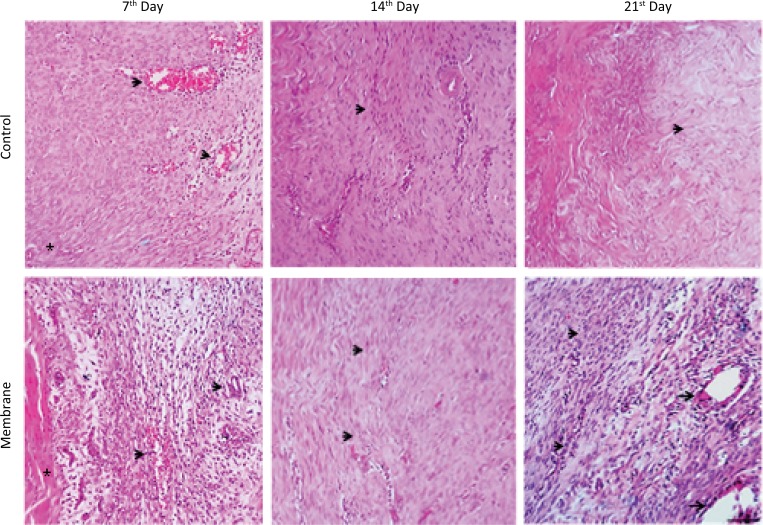
Histopathological features of the tendon repair in the control (C) and membrane (M) groups on days 7, 14 and 21 (Hematoxylin & Eosin, x160). **C1** - Control group (7 days): Well-developed granulation tissue with newly formed congested blood vessels (*arrows*) and young fibroblasts near the residual tendon (*); **M1** - Membrane group (7 days): newly formed blood vessels (*arrows*), young fibroblasts near the preserved residual tendon (*); **C2** - Control group (14 days) and M2 - Membrane group (14 days): thin fibroblasts arranged in compact bundles (*arrows*); **C3** - Control group (21 days): very dense and hyalinized extracellular matrix (*arrow*); **M3** - Membrane group (21 days): mature fibroblasts (*smaller arrows*) arranged in bundles parallel to the pre-existing tendon; granulomas (*larger arrows*).

Qualitative histological analysis of the control group on day 7 showed that the tenotomized area was occupied by granulation tissue, characterized by an edematous ECM containing newly-formed blood capillaries, macrophages, and lymphocytes. The neutrophils were scarce and fibroblasts, which had an abundant cytoplasm and large nucleus, were generally located parallel to the preserved tendon and the most compact ECM in the areas closest to the uninjured tendon (Fig. 1, C1). Analysis of the membrane group showed granulation tissue with a very loose and swollen ECM containing macrophages, lymphocytes, and polymorphonuclear neutrophils. Newly-formed, congested, thin-walled blood vessels were abundant and fibroblasts were concentrated in a stripe near the preserved tendon (Fig. 1, M1).

#### Fourteen-day groups

On day 14 post-injury, the granulation tissue of the control group had a denser ECM containing fibroblasts with thin and wavy nuclei arranged in compact bundles. Blood vessels and inflammatory cells were scarce (Fig. 1, C2). In the membrane group, the granulation tissue had a very dense ECM with infrequent inflammatory cells and few newly-formed blood vessels. The fibroblasts were located parallel to the preserved tendon (Fig. 1, M2).

#### Twenty-one-day groups

On the 21st day after tenotomy, the control group had a very dense hyaline-like ECM with few blood vessels and inflammatory cells. Fibroblasts, which had thin and tortuous nuclei, were arranged in poorly-organized bundles (Fig. 1, C3). In the membrane group, granulation tissue had a dense ECM with few blood vessels and fibroblasts arranged in bundles parallel to the residual tendon. Inflammatory cells were scarce but granulomas were found around optically-empty spaces or filled with amorphous or fibrillar material composed of macrophages and foreign body giant cells (Fig. 1, M3).

### Analysis of collagen fibers

**Figure 2:**
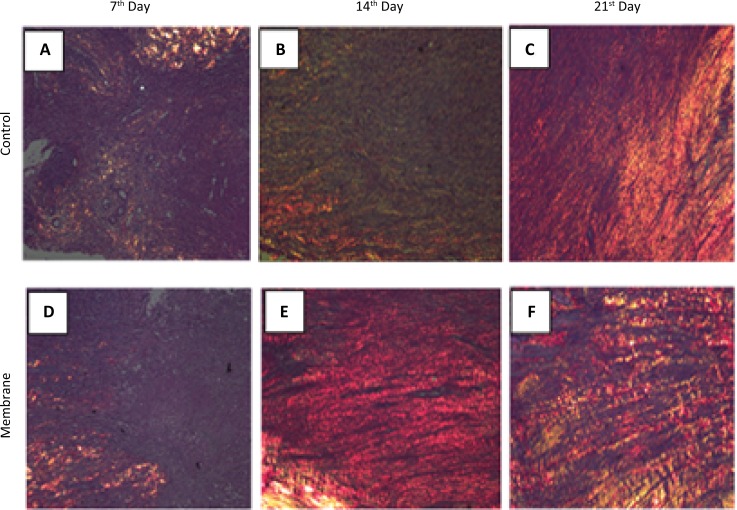
Qualitative features of control and membrane collagen fibers stained with Picrosirius Red (x10).

Qualitative analysis of collagen fibers was performed by observing slides stained with Picrosirius Red, which showed the gradual replacement of type III with type I collagen. On day 7 post-injury, green fibers were predominant, which represent type III collagen, both in the control and membrane groups, as shown in Figure 2A and D. On day 14, the control group still had a high concentration of type III collagen (Fig. 2B), whereas the membrane group had mostly type I fibers, which were represented as red fibers (Fig. 2E). On day 21, the histological sections of the control group tendon lesion also showed the pronounced presence of type III collagen, although type I collagen was also present, as shown in Figure 2C. Type III collagen was still present in the membrane group, but in a slightly larger proportion compared to that in the type I collagen group (red fibers; Fig. 2F).

### Quantitative analysis

The intragroup analysis of the control showed that there was a significant decrease in the mean inflammatory cell count in the periods of 7-21 days (p=0.0004) and 14-21 days (p=0.0004). The membrane group also showed a significant reduction in the periods of 7-21 days (p=0.0001) and 14-21 days (p=0.0010) (Table 1).

**Table 1:** Intragroup analysis of the mean inflammatory cell count (mean ± standard error).

Groups	Period (days)			IGA		
	7	14	21	p (7 vs 14)	p (7 vs 21)	p (14 vs 21)
Control	*160.3 ± 4.56*	*161.1 ± 6.06*	*115.5 ± 9.35*	*ns*	* *0.0004*	* *0.0004*
Membrane	*94.35 ± 2.13*	*85.15 ± 2.13*	*82.65 ± 0.87*	*ns*	* *0.0001*	* *0.0010*

IGA = intragroup analysis;

* = significant difference;

ns = not significant difference

**Figure 3:**
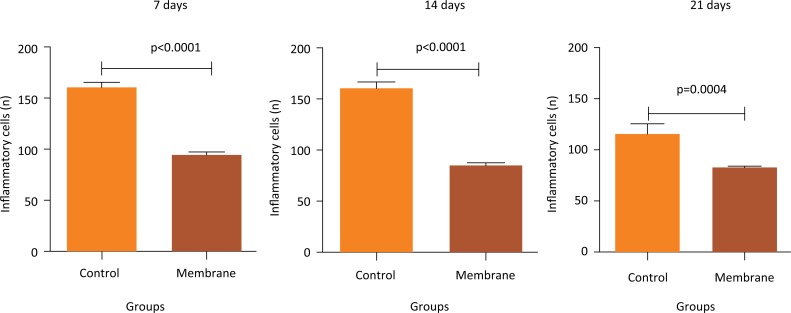
Intergroup analysis of the mean number of inflammatory cells on day 7, 14 and 21 of the experiment.

Intergroup analysis of mean inflammatory cell counts (Fig. 3) showed that the control group had the highest values for all analyzed periods (7, 14, and 21 days). Further, on days 7 and 14, the membrane group showed an extremely significant reduction compared to values in respective control groups (p < 0.0001). On day 21, cell counts in the membrane group were still markedly lower compared to those in the control (p = 0.0004).

Table 2 shows the intragroup analysis of the mean number of fibroblasts. For this, compared to that at 7 days, the control group showed a significant increase at 14 (p = 0.0007) and 21 days (p = 0.0002). In contrast, compared to that at 7 days, the membrane group showed a significant reduction in the number of fibroblasts at 14 (p = 0.0041) and 21 days (p = 0.004).

**Table 2:** Intragroup analysis of the fibroblast count (mean and ± standard error) on day 7, 14, and 21 of the experiment.

Groups	Period (days)			IGA		
	7	14	21	p (7 vs 14)	p (7 vs 21)	p (14 vs 21)
Control	*55.96 ± 2.64*	*72.00 ± 2.67*	*77.4 ± 4.18*	* *0.0007*	* *0.0002*	* *ns*
Membrane	*107.60 ± 2.64*	*98.46 ± 2.16*	*99.55 ± 3.20*	* *0.0041*	* *0.0041*	*ns*

IGA = intragroup analysis;

* = significant difference;

ns = not a significant difference

**Figure 4:**
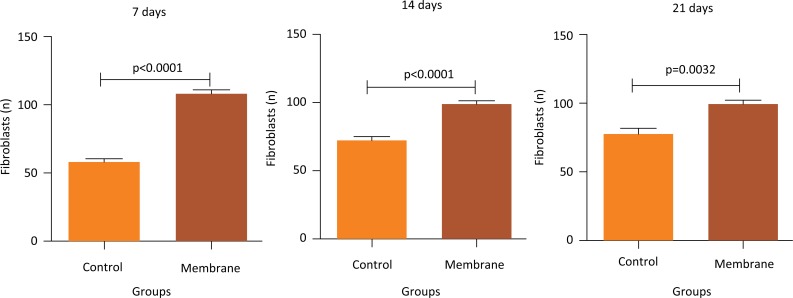
Intergroup analysis of the mean number of fibroblasts on days 7, 14, and 21 of tendon repair in rats comparing control and membrane groups.

Intergroup analysis of the mean fibroblast counts showed that the membrane group had the highest mean at all time points (7, 14, and 21 days). On days 7 and 14, mean counts in the control group were markedly and significantly reduced compared to those in the membrane group (p < 0.0001). Further, on day 21, the control group still showed a significant reduction compared to that in the membrane group (p = 0.0032) (Fig. 4).

Intragroup analysis of collagen deposition in the control group showed an increase in the percentage of type I collagen fibers and a decrease in type III fibers over time and the difference was significant between 7 and 21 days for both (p = 0.0012 and p = 0.0029, respectively). The membrane group also showed an increase in the percentage of type I fibers from 7 to 14 days and a slight decrease from 14 to 21 days, with a decrease in the percentage of type III fibers from 7 to 14 days. However, the differences were not significant comparing all time points. Table 3 shows the intragroup analysis of the control and membrane groups with mean and standard error.

**Table 3:** Intragroup analysis of type I and type III collagen count (mean and ± standard error) on day 7, 14 and 21 of the experiment.

Groups / TC (%)	Period (days)				IGA		
		7	14	21	p (7 vs 14)	p (7 vs 21)	p (14 vs 21)
Control	CTI	*2.66 ± 0.65*	*20.49 ± 6.19*	*32.73 ± 7.54*	*ns*	* *0.0012*	*ns*
	CTIII	*95.08 ± 2.32*	*79.51 ± 6.19*	*67.27 ± 7.54*	*ns*	* *0.0029*	*ns*
Membrane	CTI	*25.71 ± 6.28*	*51.35 ± 9.37*	*41.70 ± 5.98*	*ns*	*ns*	*ns*
	CTIII	*70.09 ± 7.17*	*49.19 ± 9.19*	*57.17 ± 6.26*	*ns*	*ns*	*ns*

TC = types of collagen; CTI = collagen type I; CTIII = collagen type III; IGA = intragroup analysis;

* = significant difference (p < 0.05);

ns = not a significant difference'

**Figure 5:**
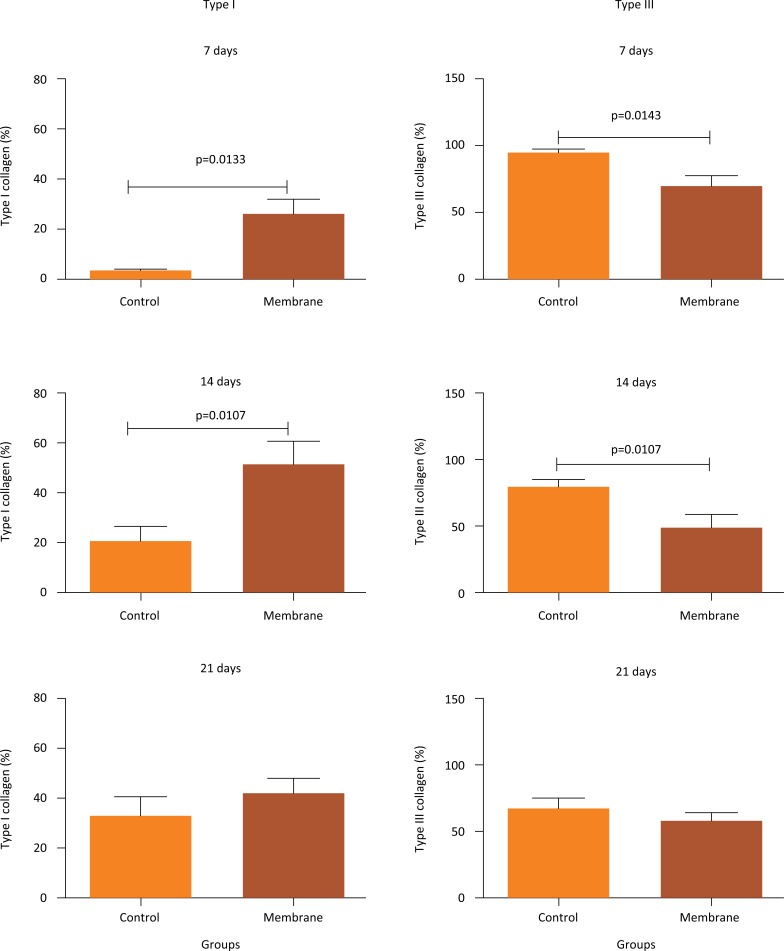
Intergroup analysis (mean ± standard error) of type I and type III collagen deposition on days 7, 14, and 21 of tendon repair in rats comparing control and membrane groups.

Intergroup analysis showed that the membrane group had a significant increase in type I collagen fibers on days 7 (p = 0.0133) and 14 (p = 0.0107) compared to those in the controls. Further, the analysis of type III collagen fibers showed a significant decrease in the membrane group on days 7 (p = 0.0143) and 14 (p = 0.0107) compared to levels in the control groups. On day 21, there were no significant differences between groups (Fig. 5).

## Discussion

Considering the previously reported therapeutic properties^12^ ^,^ ^16^ of PHB and norbixin, which comprised the membrane used in the present study, and the rate of Achilles tendon rupture that reduces the function of the ankle joint, we decided to evaluate the efficacy of this biomaterial with respect to the inflammatory response and organization of collagen fibers at different stages during the healing process after tenotomy. According to Alves *et al.* ^21^ and Monte *et al.* ^15^, biocompatible membranes are widely applied in health care to promote tissue repair; the main biological principle of this type of treatment is guided regeneration in which a membrane should favor the maintenance of stimulating factors for tissue repair, function as a cell scaffold for proliferation, and prevent the invasion of soft tissues in the injured area, thus preventing heterotrophic cell inhibition, to be considered ideal. This is in addition to non-biological factors that include easy manufacturing and storage and low cost^22^.

Studies using norbixin-based PHB membranes, which are considered easy to manufacture and associated with low cost, have shown promising results in animal models in addition to meeting adequacy and safety standards^12^ ^–^ ^16^. Monte *et al.* ^15^ evaluated the mutagenic potential of PHB membranes through a micronucleus test and comet assay in rats using bone marrow. They showed that the membrane did not exert any genotoxic effects. In an *in vivo* rat study by Sousa *et al.* ^13^, an analysis of the genotoxicity of norbixin- and ethylene glycol-based PHB membranes showed that damaged DNA was repaired in only 24 h, which confirmed that the membrane could be used in biological tissues.

The results of this study, both qualitative and quantitative, showed that the acute inflammatory process in the control group was heightened compared to that in the membrane group, thus showing the anti-inflammatory effects of the PHB membrane with norbixin. A study by Capella *et al.* ^23^ showed the significant proinflammatory activity of an annatto (*Bixa orellana* L.) oily extract, which was associated with a favorable outcome with respect to the healing process of epithelial injury and a lower inflammatory density on day 7 compared that in the other groups. This was due to the fatty acids (linoleic and oleic) contained in the preparation, which have chemotactic properties for poly and mononuclear cells, accelerating the healing process, as shown by increased scar formation and faster epithelialization. In addition, fatty acids facilitate the action of growth factors, humidifying the environment and stimulating the formation of granulation tissue, which accelerates the healing process. In contrast, the tendon is known to be a poorly vascularized tissue, which consequently has low nutrition and oxygenation demands, and thus has a low capacity for tissue repair^24^. This accounted for the significant presence of inflammatory cells at the end of day 21.

According to Alaseirlis *et al.* ^25^ and Nicodemo *et al.* ^26^, reductions in the inflammatory response in the early stages of tendon injury repair improves tendon quality. Thus, in this study, comparing inflammation in control and membrane groups, it can be inferred that the tendons of the membrane group were of higher quality. An important finding in this study was the formation of granulomas in the membrane group. Mariani *et al.* ^27^ suggested that this foreign body reaction represents chronic inflammation comprised mainly of multinucleated giant cells and can occur in response to the implantation of a biomaterial. However, it cannot be concluded that the granulomas observed in this study were produced due to membrane features or immunological responses in recipient animals since they were not detected in the membrane groups at days 7 and 14. However, even though granulomas formed in only one sample of group M3, further studies should be performed for longer periods to determine whether norbixin-based PHB membranes cause this type of reaction. In their case study, Ergin *et al.* ^28^ showed the occurrence of granuloma formation with Achilles tendon silk suture many years after surgery. Further, Henry *et al.* ^29^ reported a case of late granulomatous formation after the use of a LARS (Ligament Advanced Reconstruction System) ligament for Achilles tendon reconstruction in humans, which shows that biomaterials that are highly reliable for clinical use, even if rarely, can also induce the formation of granulomas.

Regarding the number of fibroblasts, all animals treated with the PHB and norbixin membranes had significantly higher values compared to those in respective control group animals. Histological analysis further showed even better organization of fibroblasts in the membrane group. Fibroblasts are known to play an important role in synthesizing type III collagen in the early stages of the tissue repair process. This collagen (type III) is characterized by small and weak fibers with lower mechanical strength. Collagen type I, which is also synthesized by fibroblasts in the remodeling stage of tendon repair and exists in greater proportions in the tendon, confers strength and quality to this anatomical region. Thus, a high number of fibroblasts indicates increased collagen production in the ECM of the tendon^7^.

As expected, the production of type III collagen, which is important for healing, decreased at approximately day 14 of the repair process, as it was gradually replaced by type I collagen. Whereas type I collagen was produced, intragroup results showed that the percentage of collagen in the tissue was not in agreement with the results of other studies^7^ ^–^ ^9^ on tissue repair, as a higher amount of collagen type I (mature), and not type III (immature), was expected in the last repair stage on day 21. However, the intergroup results showed that the membrane group produced more type I collagen than the control group at all time points of the experiment. Considering these data, it is possible to infer that the presence of the membrane near the tendon injury could stimulate tissue repair, as shown by histological features in comparison to those in the control group. Another unexpected result was the slight increase in type III collagen in the membrane group on day 21 of the experiment. However, it should be noted that the formation of a foreign body granuloma occurred in this group (M3), which altered the final stage of tendon tissue repair and was likely accompanied by scar formation. Baker *et al.* ^30^ pointed out that fibroblast proliferation and collagen production are hallmarks of fibrotic tissue in reactions to biomaterial implants and can lead to increased collagen production, ECM secretion, and implant encapsulation when activated.

Considering this, it is noteworthy that the membrane used in this study, although synthetic, is easy to manufacture, associated with low cost, and easy to store. Specifically, it does not require the use of specific substances for its conservation, and these represent advantages over traditional membranes. However, as this was the first study conducted on the use of this biomaterial for tendon repair, further studies are required to characterize other properties with respect to its application to tissue injuries and its prospects for clinical use.

## Conclusions

The application of a PHB membrane with norbixin after total tenotomy of the Achilles tendon in rats favored the tissue repair process, as it reduced the inflammatory response, stimulated fibroblast proliferation and, consequently, collagen neoformation. However, we emphasize the importance of further studies to observe the interactions between this membrane and the tendon tissue, which could thus attest to the feasibility of using this biomaterial clinically for acute tissue injuries.
